# Supplementary material for: How Does the Family Influence Adolescent Eating Habits in Terms of Knowledge, Attitudes and Practices? A Global Systematic Review of Qualitative Studies
Source: Nutrients. 2021 Oct 22;13(11):3717. doi: 10.3390/nu13113717 (PMC8624651; doi:10.3390/nu13113717)
Supplement: Supplementary file 1 [file nutrients-13-03717-s001.zip › nutrients-1408289-supplementary.pdf]

Table S1. Data extraction of reviewed studies

**Study**	**Research design**	**Sample characteristics**	**Major findings**		
Author, year, country of origin Aim(s)	Data collection method, setting, source of data, analytical method	Sample size, age and sex of adolescents (other informants), sampling method	Family factors of adolescent KAP<br><br>Knowledge	Attitudes	Practices
Backett-Milburn, 2006, UK [43]<br><br>To examine the perceptions and understandings underpinning the dietary practices in families with 'normal' weight and 'overweight' young teenagers living in poorer socio-economic circumstances	DI<br><br>Home setting in socio-economically disadvantaged areas in Eastern Scotland<br><br>Reported by parents/main food providers<br><br>Inductive thematic analysis	*N* = (34)<br><br>Aged 13-14 years<br><br>Female = (97%)<br><br>Purposive sampling by gender, BMI, and sociodemographic details		Family motivation<br>Parents not associating fatness in early teens as health risk and emphasizing to their child that weight should not be a worry or concern for them *(B)*<br><br>Viewing the food choice and eating practices as the teenagers' own responsibility *(B)*<br><br>Dietary issues being of a low priority in the hierarchy of risk and worry facing their teenagers, e.g. friends, activities, school work, and other health-damaging issues including drugs, smoking, alcohol and sex *(B)*<br><br>Issues around body shape and size at this age was less potentially problematic than the risks to teenagers' mental or physical health of becoming obsessed with weight loss, and over-concern might be counter-productive *(B)*	Food preparation and availability<br>Parents providing healthy foods at home *(F)*<br><br>Parenting style<br>Parents setting limits on the teenager's consumption of junk and snack foods *(F)*<br><br>Parents accommodating children's taste preference without insisting them to eat disliked healthy foodstuffs *(B)*<br><br>Parents accommodating varied tastes simultaneously, coordinating schedules, keeping everyone happy and ensuring food was not wasted by convenience and snack foods *(B)*

Backett-Milburn, 2010, UK [44]<br><br>To understand more about the social and cultural conditions which might be promoting more positive dietary health and physical well-being amongst middle class families	DI<br><br>School setting in relatively advantaged areas in Eastern Scotland<br><br>Reported by parents<br><br>Not specified (Inductive thematic analysis)	*N* = (35)<br><br>Aged 13-14 years<br><br>Female = (94%)<br><br>Purposive sampling by sociodemo graphic details	Nutrition education<br>Parental education on the nutritional need (e.g. a wide variety of food, vitamins) and healthy food choice *(F)*	Cultivation of food preference<br>Healthy food provisioning to cultivate and manipulate dietary tastes, e.g. cooking from scratch/basic ingredients with minimal reliance on pre-prepared ingredients, fast foods or ready meals *(F)*<br><br>Parents encouraging the development of a varied and nutritional 'adult' diet and cosmopolitan tastes *(F)*<br><br>Parents counterbalancing the temptation of junk food with 'activities' *(F)*<br><br>Family motivation<br>Parents setting a good example themselves *(F)*	Food preparation and availability<br>Parents limiting snacks purchase and availability *(F)*<br><br>Time and cost<br>Requiring time and effort and not unproblematical for the 'from scratch' food preparation *(B)*<br><br>Busy schedule of both adolescents and parents for family home meals *(B)*<br><br>Parenting style<br>Parental control and monitoring of the food their children eat at home, e.g. controlling portion sizes by serving the meals onto the plates for their families, and/or commenting to their teenager if they felt s/he either ate too much or did not take adequate amounts of vegetables *(F)*<br><br>Parental inclusion and acceptance of a modest amount of these 'unhealthy' foodstuffs instead of total forbidding *(F)*<br><br>Parental practical knowledge and attitudes<br>Hiding fruit and vegetables in soups or stews *(F)*
Banna et al, 2016, Peru [39]<br><br>To understand socio-cultural influences on eating among adolescents in periurban Lima, Peru using qualitative methods	DI<br><br>Home setting in low-income Spanish-speaking district<br><br>Reported by adolescents<br><br>Direct	*N* = 14<br><br>Aged 15-17 years<br><br>Female = 43%<br><br>Purposeful random sample from census	Nutrition education<br>Family members stressing the importance of healthy, regular meals for disease prevention *(F)*<br><br>Parents providing advice on food selection, such as certain food groups and a variety of foods, and fostering cooking skills to help adolescents make healthy food choices *(F)*	Family motivation<br>Family members encouraging adolescents to consume foods prepared at home rather than purchased from street vendors *(F)*<br><br>Family members stressing the importance of healthy, regular meals for disease prevention *(F)*	Food preparation and availability<br>Parents providing home-cooked meals *(F)*<br><br>Time and cost<br>Lack of financial resources to purchase healthy foods *(B)*

	content analysis		Child involvement<br>Adolescents who helped mothers prepare dishes being able to list ingredients and quantities in recipes used *(F)*		
Bassett, 2008, Canada [45]<br><br>To explore how adolescents and parents negotiate adolescents' increasing food choice autonomy in European Canadian, Punjabi Canadian and African Canadian families	DI, participant observation at a family meal and a grocery shopping trip with the family shopper(s)<br><br>Home setting<br><br>Reported by related adolescents and parents separately<br><br>Thematic and constant comparative analyses	*N* = 47 (62) from 36 families<br><br>Aged 13-19 years (mean = 41 years)<br><br>Female = 72% (68%)<br><br>Snowball sampling	Nutrition education & Family illness experience<br>Mothers invoking health discourses, e.g. family health concerns, to explain food choices to their teenage children and coach them towards specific choices *(F)*	Family motivation<br>Teens monitoring their own food choices by watching and listening to their mothers, aunts and grandmothers *(F)*<br><br>Parents using nagging to encourage their teenage children to eat in particular ways, e.g. making their supper or eating in healthy ways *(F)*	Food preparation and availability<br>Parents controlling household food supplies by purchase and meal preparation *(F)*<br><br>Parenting style<br>Parents using various strategies to coerce adolescents to eat foods they disliked, ranging from providing a number of choices from which to choose, to insisting teens take a bite or two of the offending food *(F)*<br><br>Parents not encouraging nor allowing teenagers to accompany them on shopping trips to prevent from selecting unhealthy food items *(F)*

Berge et al, 2012, USA [36]<br><br>To explore multiple family members' perceptions of risk and protective factors for healthy eating and physical activity in the home.	Multi-family FG<br><br>Community setting<br><br>Reported by adolescents and family members, jointly<br><br>Grounded hermeneutic approach	*N* = 70 (33) from 26 families<br><br>Aged 8–12, 13–18 and ≥19 years (26-61 years)<br><br>Female = 54% (67%)<br><br>Voluntary response sampling		Family health<br>Parental modeling to identify the positive feelings that are associated with being healthy *(F)*<br><br>Cultivation of food preference<br>Making healthful eating fun (e.g. during grocery shopping) *(F)*<br><br>Family motivation<br>Spreading individual investment in being healthy to other family members, e.g. among siblings *(F)*<br><br>Participating in healthful behaviors together (e.g. going shopping) and supporting family members when they were trying new things to be healthful *(F)*<br><br>Making healthful eating routine and plan for it to happen, making it part of their family lifestyle *(F)*	Food preparation and availability & Parenting style<br>Involving the whole family in eating more healthfully through routines such as family meals *(F)*<br><br>Time and cost<br>Parents' work hours, children's and adolescent's schedules, the time it takes to prepare meals and having too many obligations contributed to the difficulty in eating healthfully *(B)*<br><br>High cost of healthy options over fast food restaurants, e.g. McDonald's or a Wendy's or Burger King *(B)*
Brown et al, 2015, Botswana [46]<br><br>To describe the factors that influence adolescent and adult perceptions and attitudes related to adolescent diet in Botswana	FG<br><br>School setting<br><br>Reported by unrelated adolescents and parents<br><br>Not specified (low level, non-abstract, direct interpretation)	*N* = 12 (3) groups<br><br>Aged 12-18 years<br><br>Female = 6 groups (not specified)<br><br>Purposive sampling by SES			Food preparation and availability<br>Home restrictions to healthy food choices by food availability (reported by adolescents) *(F)*<br><br>Time and cost<br>Limited time and financial resources for the preparation of healthy foods comparing to the expediency of fast food (reported by parents) *(B)*

Calvert et al, 2020, UK [47]<br><br>To explore the perceptions of healthy eating behaviours and the influences on eating behaviours amongst 11-to-13-year-old secondary school students	Semi-structured same-sex FG<br><br>Schools located in deprived areas of England<br><br>Reported by adolescents<br><br>Thematic framework analysis	*N* = 46<br><br>Aged 11-13 years<br><br>Female = 54%<br><br>Purposive sampling of selected community (low-income)		Family motivation<br>Negative impact of unhealthy family lifestyle and culture *(B)*	Food preparation and availability<br>Lots of unhealthy food but no, or few, healthy options available to them at home *(B)*<br><br>Time and cost<br>Unhealthy food being cheaper compared to healthy foods *(B)*
Chan et al, 2009, HK [34]<br><br>To explore Chinese adolescents' perceptions of healthy eating, their perceptions of various socializing agents shaping their eating habits, and their opinions about various regulatory measures which might be imposed to encourage healthy eating	FG<br><br>Low to middle class population<br><br>Reported by adolescents<br><br>Comparison analysis method	*N* = 22<br><br>Aged 13-15 years<br><br>Female = 41%<br><br>Purposive sampling of selected community	Nutrition education<br>Being warned by parents and family members about the undesirable consequences of unhealthy eating, such as heart problems and kidney malfunction *(F)*		Food preparation and availability<br>Adolescents being more likely to practice healthy eating at home; foods prepared at home having less sugar, oil and salt, and being MSG free *(F)*<br><br>Parenting style<br>Mothers placing restrictions on the quantity of unhealthy foods consumed at home *(F)*

Christiansen et al, 2013, USA [48]<br><br>To understand environmental factors influencing the food-related habits of low-income urban African American adolescents	DI, FG sessions, and direct observation<br><br>Baltimore City Recreation Centers located in low-income predominantly African-American neighborhoods participating in Baltimore Healthy Eating Zones (BHEZ)<br><br>Reported by adolescents<br><br>Thematic analysis	*N* = 20<br><br>Aged 10-16 years<br><br>Female = 75%<br><br>Stratified, purposive sampling	Nutrition education & Child involvement<br>Open discussions on the negative health outcomes associated with unhealthy eating *(F)*<br><br>Family illness experience<br>Exposure to family health history within the social environment as an important influence on how young people thought about the relationship between food and health *(F)*	Family health<br>Family histories of obesity, diabetes, and heart disease motivating adolescents to consume food identified as healthy, such as fruits and vegetables *(F)*<br><br>Family motivation<br>Attitudes toward foods, especially fruits and vegetables, being shaped by attitudes expressed by role models (e.g. family members) *(F/B)*	Food preparation and availability<br>Parents primarily impacting the availability of food in the home and the food purchased by adolescents when a role model is present *(F)*<br><br>Parenting style<br>Parental monitoring adolescents' food consumption and establishing rules that restrict or permit the food choices they make *(F)*
Correa et al, 2017, India and Canada [49]<br><br>To understand perceptions and attitudes of Indian-origin adolescents in	FG<br><br>School setting in rural and urban India, and urban Canada<br><br>Reported by adolescents	*N* = 73<br><br>Aged 11-18 years<br><br>Female = 53%<br><br>Stratified, purposive sampling	Nutrition education<br>Parents providing guidance and education on making healthy choices *(F)*	Family motivation<br>Watching sibling eat unhealthy foods increased cravings for them *(B)*	Food preparation and availability<br>Parents preparing and purchasing healthy foods *(F)*<br><br>Availability and easy accessibility of unhealthy foods at home or near home *(B)*<br><br>Time and cost<br>Parents lacking the time to prepare meals at home because of long working hours so they ate store bought food *(B)*

India and Canada that may contribute to healthy eating behaviour	Thematic analysis with framework approach				Having money, and affordable pricing of unhealthy foods made it easy to acquire and consume unhealthy foods by adolescents *(B)*<br><br>Parenting style<br>Parents providing mealtime supervision *(F)*
Cullen, 2002, US [50]<br><br>To assess social-environmental influences on children's FJV and low-fat choices	FG by ethnicity<br><br>School setting among low-income families<br><br>Reported by adolescents and parents separately (relationship not specified)<br><br>Not specified	*N* = 180 (40)<br><br>Aged 9-12 years (mean = 36, 41, 44 years)<br><br>Female = not specified (91.7-100%)<br><br>Voluntary participation		Family motivation<br>Parents encouraging their children to eat FJV *(F)*<br><br>Lack of parent examples (both their own and in general) as a reason children did not eat FJV *(B)*	Food preparation and availability<br>Availability of a variety of fresh FJV, and some canned or frozen FJV, in the homes, or not *(F/B)*<br><br>Time and cost<br>Importance of accessibility, e.g. cutting up *(F)*<br><br>Cost of FJV out of season and of 100% juice as the major reasons for not purchasing them and not having them available at home *(B)*<br><br>Parenting style<br>Parents insisting that the children try at least one bite of FJV by setting rules e.g. requiring them to finish all the foods on their plate *(F)*<br><br>Parents monitoring child growth and health by asking what foods their children consumed during the day, and a great concern about weight and potential problems with obesity *(F)*
Darling, 2015, US [40]<br><br>To gain a deeper and more insightful understanding of the family	DI<br><br>School setting<br><br>Reported by parents	*N* = (29)<br><br>Grade 10, appx aged 15 years (35-60 years)<br><br>Female: (86%)	Child involvement<br>Discussion to provide guidance on healthy eating, e.g. junk food, sugar added to drinks *(F)*<br><br>Adolescents having minimal responsibility for shopping, food	Family health<br>Understanding how important it is to choose the right things to eat and being "health conscious" by having family members with health conditions, e.g. arthritis and diabetes *(F)*<br><br>Family motivation<br>Parents modeling poor health habits (i.e.	Food preparation and availability<br>Eating fast food and takeout food (e.g., pizza, chicken wings) for home meal at least one or two times a week *(B)*<br><br>Time and cost & Parenting style<br>Rationalizing the unhealthy eating habits developed by blaming circumstances such as divorce, grandparents in the home who "spoiled"

food ecosystem, parental modeling, and parental indulgence and their potential relationship to adolescent health and well-being	Content analysis	Voluntary response sampling	preparation, cleaning up, or other related tasks necessary for healthy meals due to pressures in their lives, e.g. homework, sports, and other immediate personal or social needs *(B)*<br><br>Family illness experience<br>Some adverse conditions of family members making 'food' or 'heath' a 'sensitive issue' for discussion *(B)*	crash diets, irregular meals and eating fast food and junk food) *(B)*	their children, "independent" children, or busy schedules *(B)*<br><br>Parenting style<br>Inconsistent rules and contradictory behaviors in the family, e.g. not buying sugary drinks to home but eating fast food for family meals *(B)*<br><br>Lack of monitoring of food choices both at school and home with access to both healthy and unhealthy food as parents equating with trusting their adolescents to eat appropriately *(B)*

Fielding-Singh, 2017, USA [51]<br><br>To examine an overlooked contributor to family diet: fathers	DI<br><br>Home setting in middle or upper class families<br><br>Reported by related adolescents and parents separately<br><br>Qualitative content analysis	N = 53 (56) from 44 families<br><br>Aged 12-19 years<br><br>Female = not specified (75%)<br><br>Purposive sampling by family characteristics of interest		Family motivation<br>Fathers modeling unhealthy dietary behaviors to adolescents, and feeding adolescents unhealthy foods (reported by mothers) *(B)*<br><br>Fathers caring less about the healthiness of their children's diets, seeing health and nutrition as the mother's domain (reported by fathers) *(B)*	Food preparation and availability<br>Purchasing and preparing healthy foods by mothers (reported by mothers) *(F)*<br><br>Time and cost<br>Lack of time for the preparation of healthy home meals and going for takeout food choices (reported by parents), where fathers readily turning to quick, unhealthy options out of convenience or a lack of provisioning skills (reported by both) *(B)*<br><br>Parenting style<br>Mothers regulating unhealthy food intake (reported by adolescents) *(F)*<br><br>Little intervention in adolescents' unhealthy food practices (reported by fathers) *(B)*<br><br>Provision of junk food by fathers due to the lack of paternal concern of a healthy diet (reported by both) *(B)*<br><br>Parental practical knowledge and attitudes<br>Paternal neglection of nutritional values during grocery shopping and being incapable of purchasing the foods necessary to keep her children healthy (reported by mothers) *(B)*<br><br>Provision of junk food by fathers due to the lack of paternal concern of a healthy diet (reported by both) *(B)*

Fielding-Singh and Wang, 2017, USA [52]<br><br>To explore how mothers and adolescents across SES talk about food	DI<br><br>Community setting across socioeconom ic status<br><br>Reported by related adolescents and parents separately<br><br>Qualitative content analysis	N = 62 (62) from 62 families<br><br>Aged 12-19 years<br><br>Female = 61.3% (100%)<br><br>Purposive and theoretical sampling by socioeconomic status	Nutrition education<br>Framing concerns about their adolescent's weight as concerns about eating healthy and being healthy, potentially avoiding negative consequences of discussing weight with their adolescents (reported by high-SES mothers) *(F)*<br><br>Child involvement<br>Conversations about healthy eating were commonplace within family life, occurring regularly during mealtimes, shopping trips, and restaurant outings (reported by both from high-SES) *(F)*<br><br>Not modeling their own ideals of healthy eating, which they believed limited their ability to talk to their adolescents about healthy eating (reported by low-SES mothers) *(B)*	Cultivation of food preference<br>Conversations about healthy eating as a critical part of molding their adolescents' preferences, by, e.g. using dialogue to explain and justify to their adolescents their dietary standards (reported by high-SES mothers) *(F)*<br><br>Understanding from discussions with their mothers that their family diet was less healthy because they could not afford the high cost of healthy eating (reported by low-SES adolescents) *(B)*<br><br>Family motivation<br>Viewing discussions of healthy eating as an important component of setting expectations about diet, guiding their choices, or instilling lessons to help them make healthier choices on their own (reported by both from high-SES) *(F)*<br><br>Few conversations about healthy eating as parents not regarding food as a central topic of discussion, and the healthiness of their adolescents' consumption was not a top concern (reported by low-SES mothers) *(B)*	Time and cost<br>Price as a constraint on their ability to purchase the highest quality food, and trade-offs between the cost, quality and the healthiness of food (reported by middle-and low-SES families) *(B)*<br><br>Parenting style<br>Conversations about healthy eating not always translating into enforced rules around food, e.g. saying "you should stop eating this" but not taking it away from them (reported by low-SES adolescents) *(B)*<br><br>Parental practical knowledge and attitudes<br>Their mothers' concerns for health and food quality as being the most important factors in food purchases (reported by high-SES adolescents) *(F)*

Fitzgerald et al, 2010, Ireland [53]<br><br>To explore children's and adolescents' perspectives on the potential individual, social, environmental and developmental factors that influence their food choices.	FG<br><br>Primary and secondary schools<br><br>Reported by adolescents<br><br>Inductive thematic analysis	N = 29<br><br>Aged 9-18 years, mean = 13.67<br><br>Female = 55%<br><br>Random sampling	Food preparation and availability<br>Parental restriction on unhealthy food within the home *(F)*<br><br>Time and cost<br>Parent work schedules being a barrier to family mealtimes, often led to less healthful eating *(B)*<br><br>Parenting style<br>Parents attempting to structure and control what adolescents ate by having regular mealtimes, setting rules around meals and using food as a reward or a treat *(F)*
Fuster et al, 2019, USA [37]<br><br>To examine the perceived connections between culture and food practices among Latino pre-adolescents and their parents	DI<br><br>Community setting<br><br>Reported by parent-adolescent dyads/triads jointly<br><br>Not specified (Content analysis)	N = 17 (15) from 15 families<br><br>Aged 9-12 years (37.4 ± 5.1 years)<br><br>Female = 47.1% (93%)<br><br>Purposive sampling by ethnicity and age, and convenience sampling	Food preparation and availability<br>Limiting consumption of high fat traditional food with unhealthy foods or preparation (reported by parents) *(F)*<br><br>Time and cost<br>Lower socioeconomic status being preventive for eating unhealthy foods ("junk foods") (reported by both) *(F)*<br><br>Preference for convenience food due to fast pace of life and low price (reported by parents) *(B)*<br><br>Economic and physical access to foods deemed unhealthy (reported by both) *(B)*<br><br>Parenting style<br>Extended family, like uncles and grandparents, providing foods parents perceived as unhealthy (reported by parents) *(B)*

			Parental practical knowledge and attitudes<br>Modifying recipes to include less fat or frying, or providing a side salad or fruits, along with the “unhealthy” food (reported by parents) *(F)*
Garcia et al, 2019, USA [54]<br><br>To increase understanding of the factors that influence Hispanic/Latino childhood obesity through an intergenerational lens including children, parents/caregivers, and grandparents.	Semi-structured FG<br><br>Community based organizations in Los Angeles County<br><br>Reported by adolescents<br><br>Thematic analysis	N = 22<br><br>Aged 10-17 years<br><br>Female = 52%, one unknown<br><br>Not specified	Food preparation and availability<br>Unhealthy food options in the home environment *(B)*<br><br>Time and cost<br>Long work hours and commutes preventing adults from preparing home-cooked meals, which coupled with the convenience of fast food, supporting poor eating habits (B)<br><br>Parenting style<br>Cultural norms of parents requiring children to finish everything on the plate (F)<br><br>Using unhealthy food as reward for academic performance *(B)*<br><br>Permissive parenting styles and lack of discipline leading to unhealthy eating, e.g. parents stocking freezers and refrigerators with easily prepared meals and frozen dinners for youth to 'cook' while their parents are at work *(B)*

Goh, 2009, US [55]<br><br>To explore adolescent, parent, and community stakeholder perspectives on barriers to healthy eating and physical activity, and intervention ideas to address adolescent obesity	FG and DI<br><br>School setting<br><br>Reported by related adolescents, parents and community members separately<br><br>Not specified	N = 119 (63)<br><br>Mean age = 12 years<br><br>Female = 61% (84%)<br><br>Not specified	Child involvement<br>Parents improving communication with their children about healthy eating *(F)*	Family health<br>Children being interested in the nutrition information because of exposure to family members with medical problems, including obesity *(F)*<br><br>Family motivation<br>Parents' unhealthy eating habits *(B)*	Food preparation and availability & Time and cost<br>Ease and low cost of fast food for family meals *(B)*<br><br>Parental practical knowledge and attitudes<br>Parents not knowing about district nutrition standards or that fresh fruit was offered daily in school *(B)*
Gray, 2015, Canada [56]<br><br>To examine the eating behaviours and nutrition knowledge of young women in an Ontario secondary school	DI<br><br>Private room within school<br><br>Reported by adolescents<br><br>Not specified (Thematic analysis)	N = 20<br><br>Aged 14-17 years<br><br>Female = 100%<br><br>Not specified	Nutrition education<br>Teaching their children about the consequences of unhealthy eating and its preventions *(F)*<br><br>Learning food choices from their parents *(F)*<br><br>Getting confused by the difference messages received at home and at school *(B)*<br><br>Child involvement<br>Learning food choices from their parents as part of the meal process and during grocery shopping *(F)*<br><br>Receiving and negotiating	Family motivation<br>Learning eating behaviours of their parents as part of the meal process *(F)*	Food preparation and availability<br>Purchase and home availability of healthy food but restriction on junk food, e.g. dried mango over chips, whole-wheat over white bread *(F)*<br><br>Parenting style<br>Presence of parents for healthier eating *(F)*

			messages about self-monitoring and their responsibility in self-surveillance from their parents *(F)*<br><br>Family illness experience<br>Messages received from their parents about what eating behaviours are required to address their parents' health issues *(F)*		
Gunther et al, 2019, USA [57]<br><br>To identify practices that parents use to influence early adolescents' food choices during independent eating occasions (iEOs) from parent and child perspectives.	DI<br><br>Community setting among low income families<br><br>Reported by related adolescents and parents separately<br><br>Directed content analysis	N = 44 (49)<br><br>Aged 10-13 years (26-65 years)<br><br>Female = 56.8% (89.8%)<br><br>Purposive sampling by race and ethnicity, and convenience sampling	Nutrition education<br>Teaching children the importance of healthy eating and which foods were healthy for them to instill the value of healthy eating in their children during iEOs (reported by parents) *(F)*	Family motivation<br>Setting an example for their children in terms of consumption of particular foods, following the same rules set for the child with regard to food (reported by parents) *(F)*<br><br>Reasons for limiting specific foods including parents' concerns about children, e.g. getting sick, being hyperactive and developing diabetes (reported by adolescents) *(F)*	Food preparation and availability & Time and cost<br>Controlling the availability and accessibility of foods and beverages (i.e. no preparation needed by children) (reported by parents) *(F)*<br><br>Parenting style<br>Setting rules or expectations for their children, e.g. control portion sizes, limit calories and encourage healthy eating pattern, and consequence for noncompliance/ reward for compliance (reported by parents) *(F)*<br><br>Monitoring their children's food intake by asking the child what and how much she or he had eaten, monitoring amounts of food in cupboards or the refrigerator, communicating that they were monitoring, and calling the child when they were away (reported by parents) *(F)*

Hattersley, 2009, Australia [58]<br><br>To explore adolescents' and parents' perceptions, attitudes, and interactions in regards to screen time (ST) and sugary drink (SD) consumption	Semi-structured FG<br><br>School setting in low to middle SES areas<br><br>Reported by unrelated adolescents and parents<br><br>Thematic analysis	N = 31 (32) in 9 FG<br><br>Aged 13-16 years (mean = 46 years for mothers, 43 years for fathers)<br><br>Females = 42% (63%)<br><br>Purposive for low-middle SES	Nutrition education<br>Parents reminding the adolescents of what they should be drinking *(F)*	Family motivation<br>Role modeling as a relevant issue by some adolescents and parents *(F)*<br><br>Parents' concerns on the sugar and caffeine content, as well as acidity of various drinks, and resultant impacts on dental and general health *(F)*	Food preparation and availability & Time and cost<br>Parents controlling availability or access, and providing alternatives, particularly water *(F)*<br><br>Parenting style<br>Parents reminding the adolescents of what they should be drinking *(F)*
Heidelberger and Smith, 2015, USA [59]<br><br>To pilot Photovoice methodology with low-income, urban 9- to 13-year-olds to gain insight about their food environment and to determine whether this methodology was engaging and acceptable to them	DI<br><br>Afterschool programs for youths in Supplemental Nutrition Assistance Program (SNAP)-eligible low income households<br><br>Reported by adolescents<br><br>Open coding method	N = 29<br><br>Aged 9-13 years, mean = 11<br><br>Female = 31%<br><br>Not specified	Nutrition education<br>Parents influencing children's cooking ability *(F)*	Cultivation of food preference<br>Parents introducing new foods to their children *(F)*	Food preparation and availability<br>Parents determining the types of foods available in the household *(F)*<br><br>Parental practical knowledge and attitudes<br>Parents' health influenced their food choice, e.g. cutting down salt intake for hypertension *(F)*

Holsten, 2012, US [60]<br><br>To explore children's food choices in the home with particular attention to environmental influences	DI<br><br>School setting<br><br>Reported by adolescents<br><br>Grounded theory and content analysis	N = 47<br><br>Aged 11-14 years<br><br>Female = 51%<br><br>Maximum variation sampling by race, ethnicity, household income, and BMI		Cultivation of food preference<br>Parents' communication of nutrition information influenced the interpretation of the food (i.e. healthy/junk) which informed food preferences *(F)*<br><br>Parent's requests or encouragement for the food they eat as guiding food choices since little which became a habit *(F)*<br><br>Family motivation<br>Modeling parent healthy and unhealthy eating habits *(F/B)*	Food preparation and availability<br>Mere presence of foods in their home *(F/B)*<br><br>Time and cost<br>Monetary concerns of the parents limiting food purchases, especially of non-essential food items desired by children *(F)*<br><br>Time pressure for food preparation or guide eating decisions *(B)*<br><br>Parenting style<br>Parents asking for the child's preference to inform their decision, e.g. by providing a list of options from which the child could choose *(F)*<br><br>Children being ''allowed,'' ''not allowed,'' or required to consume certain foods or drinks by their parents *(F)*<br><br>Lack of parents' presence in the home resulting in eating food from restaurants or children eating foods they preferred and requiring less effort and skill to prepare *(B)*<br><br>Use of a food reward to guide behavior, e.g. candy for cleaning the room *(B)*<br><br>Parental practical knowledge and attitudes<br>Parents with higher skill level in preparing foods usually preparing food instead of the children *(F)*<br><br>Parents' health concerns or desire to diet shifting the entire family's food consumption towards more healthy choices, sometimes related to specific diagnoses of family members *(F)*

Islam et al, 2019, Bangladesh [61]<br><br>To explore adolescents' and mothers' perception of broader sociocultural aspects that sculpt the food choices, eating habits and physical activity behaviours of adolescents in Matlab, Bangladesh	FG<br><br>Home setting in poor, rural areas<br><br>Reported by related adolescents and parents separately<br><br>Inductive thematic analysis	N = 4 (2) groups with 6-8 participants per group<br><br>Aged 14-17 years<br><br>Female = 2 groups (2)<br><br>Purposive sampling		Cultivation of food preference<br>Lack of hygienic preparation to be the most important factor rendering foods prepared and sold by vendors in the streets inferior to foods cooked at home (reported by both) *(F)*	Food preparation and availability<br>Owning fruit trees to provide fresh fruits (reported by both) *(F)*<br><br>Time and cost<br>Lack of affordability of healthy food, e.g. fruits (reported by both) *(B)*<br><br>Parenting style<br>Persuading their children to take lunch from home to school (reported by parents) *(F)*<br><br>Parental practical knowledge and attitudes<br>Lack of knowledge on how to capitalize on locally ubiquitous vegetables in diversifying the diet and making it rich in nutrients (reported by parents) *(B)*<br><br>Home-made foods failing to meet the taste preference by adolescents (reported by both) *(B)*
Kaplan et al, 2006, USA [38]<br><br>To explore how youth, parents, and grandparents discuss issues related to eating healthfully and unhealthfully and to identify intergeneratio nal strategies for educators to improve	Semi-structured FG<br><br>Community setting among low-income populations<br><br>Reported by adolescents, parents and grandparent s jointly<br><br>Not specified	N = 21 (23) from 17 families<br><br>Aged 10-13 years<br><br>Female = not specified (not specified)<br><br>Purposive site selection and voluntary response sampling	Child involvement<br>Tailoring communication style to the child, eg, talking casually and calmly to children, providing information in an easy-to-understand way, and using humor (reported by all) *(F)*	Family health<br>Health conditions of family members with diabetes, heart disease, high cholesterol, and weight problems leading to the belief that the food people eat affects their future health (reported by all) *(F)*<br><br>Cultivation of food preference<br>Having ongoing, frequent conversation about eating healthfully to instill it in the children (reported by all) *(F)*<br><br>Family motivation<br>Encouraging good nutrition through modeling (reported by parents and grandparents) *(F)*	Food preparation and availability<br>Proactively managing foods entering the house, included buying healthful foods and limiting or banning food (reported by all) *(F)*<br><br>Time and cost<br>Scheduling problems, include sports, work, and childcare schedules, leaving insufficient time to prepare meals and eat together as a family (reported by all) *(B)*<br><br>Tight budget and concerns about food cost limiting food selection, cooking, and eating practices (reported by adolescents and parents) *(B)*<br><br>Parenting style<br>Proactively managing foods, included techniques

this communicatio n.	(Content and ethnographi c analysis)		of parents serving only one meal for all, and insisting children eat at breakfast (reported by parents and grandparents) *(F)*<br><br>Actively involving children in food-related activities including meal planning, food shopping and meal preparation (reported by all) *(F)*<br><br>Encouraging good nutrition through verbal prompting, ie, eating vegetables, avoiding fatty meats, and not overeating (reported by parents and grandparents) *(F)*<br><br>Having parents set rules for eating, e.g. one snack per day (reported by all) *(F)*<br><br>Struggle over food choices, e.g. diverse food preferences in the family, children having too many choices *(B)*<br><br>Parental practical knowledge and attitudes Disagreement on appropriate food portion by different family members, e.g. amount of snacks after school (reported by parents) *(B)*

Kumar et al, 2016, USA [62]<br><br>To enable community members to discuss their perceptions of eating habits and physical activity in relation to sixth, seventh, and eighth graders, and reveal facilitators and barriers to healthy eating behaviour and physical activity engagement.	FG<br><br>Middle school among rural, limited-resources adolescents<br><br>Reported by related adolescents, parents and teachers separately<br><br>Not specified	N = 6 groups with 6 - 8 each (13)<br><br>6th grade to 8th grade<br><br>Female = not specified (not specified)<br><br>Voluntary response sampling in selected school	Nutrition education<br>Encouraging substitution of “bad foods” with “good foods” (reported by parents) *(F)*		Food preparation and availability<br>Eating at home with healthier choices made by family members (reported by adolescents) *(F)*<br><br>Time and cost<br>Cost and convenience of foods especially dining out (reported by all) *(B)*<br><br>Lack of time for preparing and planning healthier meals and traveling (reported by adults) *(B)*<br><br>Parenting style<br>Establishing nutrition rules at mealtimes (reported by all) *(F)*<br><br>Holding purchasing power (reported by all) *(F)*
Monge-Rojas et al, 2005, Costa Rica [63]<br><br>To assess the perceptions of rural and urban Costa Rican adolescents about their diet and the factors they consider significant to	FG<br><br>School setting<br><br>Reported by adolescents<br><br>Content analysis	N = 108<br><br>Aged 12-18 years<br><br>Female = unknown, both sex<br><br>Random sampling		Cultivation of food preference<br>Adopting healthful eating practices early in life would be easier for them to eat more healthfully as incorporated as the natural thing to do *(F)*	Food preparation and availability<br>Family support by food provision at home in terms of food choice and preparation methods *(F)*<br><br>Parental practical knowledge and attitudes<br>Inadequate healthy food choices within the family diet *(B)*

healthful eating.				
Neumark-Sztainer et al, 2000, USA [64]<br><br>To (1) increase our knowledge about family meal patterns of adolescents, (2) identify factors that adolescents perceive as reasons for not eating meals with their family, and (3) assess adolescents' perceptions on whether they eat more healthful foods at family meals than in other eating situations.	FG<br><br>School setting<br><br>Reported by adolescents<br><br>Content analysis	N = 141<br><br>Age mean = 12.6 years, 16 years<br><br>Female = 61%<br><br>Not specified		Food preparation and availability<br>Food availability at meals (i.e. types of food served at meals, preparation methods, homemade/ bought food) *(F/B)*<br><br>Time and cost<br>Not having family meals due to parents' busy schedules and/or absence from the home at meal time, and teenagers' busy schedules such as work schedules, sports involvement, and home-work *(B)*<br><br>Parenting style<br>Setting rules surrounding mealtimes (e.g. not able to eat candy or sugary cereal, having to have a vegetable on their plate, having to eat whatever is cooked) *(F)*<br><br>Dissatisfaction with family relations and meal-specific concerns related to their parents' and siblings' behaviors at meals, e.g. listening to mother rattle on, as reasons for not participating in family meals *(B)*<br><br>Parental practical knowledge and attitudes<br>Health and diet-related attitudes and concerns of family members, including health-related conditions of family members or early deaths of

					grandparents, influenced eating behaviors of other family members and the types of foods served at meals in their homes *(F)*<br><br>Taste or type of food served at family meals *(B)*
O'dougherty et al, 2006, US [65]<br><br>To gain insight into parents' perceptions of the food preferences of their young adolescents, and their negotiating and decision-making strategies around food purchasing and meals	FG<br><br>School setting among nonwhite and immigrant parents with generally low socioeconomic status<br><br>Reported by parents<br><br>Not specified	N = (52)<br><br>Aged 11-15 years (mean = 37.4 years)<br><br>Female: (90%)<br><br>Voluntary response sampling		Cultivation of food preference<br>Children becoming more open to variety when there were fewer food choices in the home *(F)*	Food preparation and availability<br>Controlling intake of junk food (e.g. snacks or sweets) by restricting the purchase *(F)*<br><br>Time and cost<br>Controlling intake of junk food (e.g. snacks or sweets) by keeping out of reach or in a separate or locked "junk [food] cabinet" *(F)*<br><br>Preferring a quickly prepared meal for hectic weekday schedules *(B)*<br><br>Parenting style<br>Sharing decisions over the meal between parents and children *(F/B)*<br><br>Filling the child's requests provided the child behaved, or if the item was nutritious, or low in fat/not greasy, or once a week rather than a categorical yes or no *(F)*

Ortega-Avila et al, 2019, Mexico [41]<br><br>To explore adolescents' perceptions of how the home environment promotes the intake of sugar-sweetened beverages (SSBs) and to identify the potential environmental cues that trigger SSB intake at home	DI<br><br>Home setting or in public spaces<br><br>Reported by adolescents<br><br>Thematic analysis using framework approach	N = 29<br><br>Aged 15-19 years<br><br>Female = 55%<br><br>Purposive sampling by age and SSB intake		Cultivation of food preference<br>Preference for SSBs being a family thing *(B)*<br><br>Family motivation<br>Drinking SSBs with family members being regarded as habitual behaviour *(B)*<br><br>Constant home availability of SSBs throughout the years leading them to form a habit of consuming SSBs *(B)*	Food preparation and availability<br>Home availability of SSB *(B)*<br><br>Time and cost<br>Buying bottled SSBs when there was no time to make healthier beverages *(B)*<br><br>Parenting style<br>No rule preventing them from drinking SSBs freely at home *(B)*<br><br>Parental practical knowledge and attitudes<br>SSB restrictions and home availability influenced by the level of health consciousness among the parents, some of whom trying to lose weight or having a health condition, such as diabetes (F)<br><br>Perceiving home-made SSBs, e.g. fruit-containing beverages or fruit-flavoured drinks, independent of sugar content, as healthier than artificially flavoured drinks and acceptable to have at home *(B)*
Park et al, 2014, South Korea [66]<br><br>To identify physical and social environments that influence the eating habits of adolescents living in urban South Korea	DI and FG<br><br>Schools in urban areas<br><br>Reported by parents and teachers<br><br>Not specified (Deductive thematic analysis)	N = (9)<br><br>Aged 10-16 years<br><br>Female = (not specified)<br><br>Snowball sampling	Child involvement<br>Proper nutrition education during mealtimes at home *(F)*	Cultivation of food preference<br>Early exposure to various foods, especially to the traditional plant-based Korean diet, to develop healthy eating practices, e.g. "no picky eating, no selective eating, and no strong preference to western style diet only" *(F)*	Food preparation and availability<br>Controlling the availability of certain unhealthy food items, e.g. carbonated sodas, hamburgers, and pizzas *(F)*<br><br>Time and cost<br>Full-time working mothers eating out more often and preferring to eat out on weekends because "they are too tired to cook on weekends" *(B)*

Pinard et al, 2015, USA [67]<br><br>To understand influential factors related to family feeding practices among low-income English and Spanish speaking families with school-aged children when eating food away from home (FAFH)	DI<br><br>Community setting<br><br>Reported by related parent-adolescent separately<br><br>Not specified	N=20 (20)<br><br>Aged 8-13 years, mean = 10.5 (<21-44 years)<br><br>Female = 35% (80%)<br><br>Voluntary response and snowball followed by purposive sampling by dominant language	Nutrition education<br>Educating their children about food choices and setting up parameters by providing guidance and suggestions in child's meal choice (reported by both) *(F)*<br><br>Child involvement<br>Engaging in an authoritative feeding style to encourage child to choose healthier items, e.g. describing the motives like getting sick (reported by both) *(F)*<br><br>Providing choices for their children by an authoritative parenting style, e.g. giving more free reign, teaching about self-regulation, educating the use of nutrition labels in grocery shopping *(F)*	Family motivation<br>Parental role modeling healthy dietary behaviours (reported by parents) *(F)*<br><br>Engaging in an authoritative feeding style to encourage child to choose healthier items, e.g. describing the taste of healthier foods (reported by both) *(F)*<br><br>Parental concern about their child's weight in terms of being teased, lifelong health and chronic disease risk (reported by parents) *(F)*	Food preparation and availability & Parental practical knowledge and attitudes<br>Healthier home cooking and skills involved with home cooking (reported by parents) *(F)*<br><br>Time and cost<br>Added cost of SSBs when dining out (reported by parents) (F)<br><br>Limiting SSBs at home, providing alternatives like lemonade or water or milk (reported by parents) (F)<br><br>Parenting style<br>Using negotiation with their child to get them to eat more healthily when dining out (reported by parents) *(F)*<br><br>Obesogenic feeding style of eating not together at a table and typically in front of the television being normative practice (reported by parents) *(B)*<br><br>Lenient food rules when dining out, e.g. giving their children more autonomy in selecting their meal, allowing SSBs (reported by both) *(B)*<br><br>Price preference for unhealthy kids' meals *(B)*<br><br>Using food as reward, e.g. pizza and dessert (reported by both) *(B)*
Povey et al, 2016, UK [68]<br><br>To explore the beliefs towards eating fruit and vegetables	DI<br><br>School setting in low SE group<br><br>Reported by	N = 11<br><br>Aged 9-11 years, mean = 10<br><br>Females = 73%	Nutrition education<br>Family explaining the reasons to eat fruit and vegetables, e.g. for health, for variety of diet, weight loss and growth *(F)*		Food preparation and availability & Time and cost<br>Lack of available fruit and vegetable at home due to the high cost and high intake by siblings *(B)*<br><br>Time and cost<br>Increasing accessibility to fruit and vegetables, e.g. putting foods on dinner plate and cut up,

among children aged 9 – 11 years in a primary school setting	adolescents<br><br>Inductive thematic analysis	Convenience sampling		reducing the size of FV *(F)*<br><br>Preparation to make fruits more accessible, e.g. cut up *(B)*<br><br>Parenting style<br>Mothers and/or grandmothers making sure they have access to fruit and vegetables to eat *(F)*<br><br>Need to seek permission from family members to access unhealthy foods limit intake *(F)*<br><br>Use of unhealthy snacks as reward from caretakers, e.g. chocolate from grandmother *(B)*<br><br>Parental practical knowledge and attitudes<br>Increasing accessibility to fruit and vegetables, e.g. disguising them by mixing them together or preparing in sauces or soups made it easier to eat *(F)*

Power et al, 2010, US [69]<br><br>To provide insight into the development of a comprehensive program for the prevention of adolescent obesity: the Teen Eating and Activity Mentoring in Schools project	FG<br><br>School setting from middle-class families<br><br>Reported by unrelated adolescents, parents and teachers<br><br>Not specified	N = 16 (6)<br><br>Aged 12-14 years<br><br>Female = 69% (67%)<br><br>Not specified		Family motivation<br>Parents modeling healthy/ unhealthy eating (reported by all) *(F/B)*<br><br>Parents providing opportunities and encouraging healthy eating (reported by all) *(F)*	Food preparation and availability<br>Home availability of healthy or junk food (reported by adolescents) *(F/B)*<br><br>Time and cost<br>Hectic nature of student and family schedules, e.g. parents' jobs, children's homework, music lessons, etc leading to families eating out and eating poorly, e.g. eating prepackaged and junk foods at home (reported by all) *(B)*<br><br>Barrier of convenience, e.g. fast foods (reported by parents and teachers) (B)<br><br>Parenting style<br>Parents setting limits on junk food consumption (reported by all) *(F)*<br><br>Poor parenting, e.g. not monitoring their children, parents being permissive or inconsistent and parents overscheduling their children's lives, contributing to unhealthy behaviors, e.g. reliance on fast, takeout, and prepackaged foods (reported by teachers) *(B)*

Rakhshanderou et al, 2014, Iran [70]<br><br>To explore the determinants of fruit and vegetable consumption among Tehranian adolescents in 2012	DI<br><br>School setting<br><br>Reported by adolescents<br><br>Qualitative content analysis	N = 31<br><br>Aged 11-14 years<br><br>Female = 48%<br><br>Convenience sampling		Family health<br>Observing the intake of fruits and vegetables of parents, family members, relatives, and friends, as well as experiencing the positive outcome of fruit and vegetable consumption among the entourage *(F)*<br><br>Family motivation<br>Obeying parents'/ elders' orders as well as the existent rules at home mostly for fruits and vegetables given the health benefits *(F)*<br><br>Verbal encouragement from parents, family members, and relatives as an important motivational factor, whereas parents being peeved as a negative reinforcement *(F)*<br><br>Eating confiture and junk foods by their father/other family members being the reason for less intake of fruits and vegetables among them *(B)*	Food preparation and availability<br>Availability and accessibility of fruits and vegetables in home provided by parents *(F)*<br><br>Existence of unhealthy materials in the home as one of the barriers to consume fruits and vegetables *(B)*<br><br>Time and cost<br>Accessibility of fruits and vegetables in home provided by parents, e.g. barking or slicing fruits, exposing them to fruits and vegetables, and serving vegetables with meals *(F)*<br><br>Less availability of vegetables in their home as preparing vegetables is difficult and needs more time than fruits *(B)*<br><br>Economic factors being a barrier for the intake of fruits and vegetables *(B)*<br><br>Engagement of parents outside the home causing them not to have enough time to buy and prepare vegetables at home *(B)*<br><br>Parenting style<br>Obeying parents'/ elders' orders as well as the existent rules at home mostly for fruits and vegetables, sometimes for unhealthy food *(F/B)*<br><br>Supervision of mothers in home as they attend and obey their mothers' words and orders due to interest *(F)*

Rathi et al, 2016, India [71]<br><br>To investigate adolescents', parents', teachers', and school principals' perceptions of the main influences on adolescent eating behaviours	DI<br><br>School setting<br><br>Reported by related adolescents, parents, teachers and principals separately<br><br>Thematic analysis	N = 15 (15)<br><br>Aged 14-15 years<br><br>Female = 67% (93%)<br><br>Purposive sampling for participating schools	Nutrition education<br>Verbal encouragement on healthy food choice by authoritative parents explaining why they should have healthy foods (reported by all) *(F)*	Cultivation of food preference<br>Helping their teenagers experience a wide variety of nutritious foods by shopping for and preparing healthy foods for them to eat (reported by adolescents and principals) *(F)*<br><br>Family motivation<br>Parental role modeling of healthy eating (reported by all) *(F)*	Food preparation and availability<br>Shopping for and preparing healthy foods for their teenagers to eat (reported by adolescents and principals) *(F)*<br><br>Limiting purchase of nutrient-poor foods by parents (reported by adolescents and principals) *(F)*<br><br>Time and cost<br>Unable to prepare wholesome meals for the family and hence resort to convenience, processed foods due to time constrains among parents (reported by teachers) (B)<br><br>Parenting style<br>Negotiating which foods should be consumed by authoritative parents, e.g. avoiding fried foods (reported by all) *(F)*<br><br>Using stringent practices such as coercion to make their adolescent children eat nutritious food, e.g. drinking one glass of milk every morning (reported by all) *(F)*<br><br>Stopping them from having junk food would make them want to have that more (reported by parents) *(B)*

Rawlins et al, 2013, UK [72]<br><br>To explore both individual and family perceptions, intentions and beliefs relating to healthy lifestyles	FG and DI<br><br>School and community setting<br><br>Reported by related adolescents and their parents separately<br><br>Thematic analysis	N = 70 (43)<br><br>Aged 8-13 years<br><br>Female = 56% (79%)<br><br>Purposive sampling by ethnicity and age	Child involvement<br>Involving children to use food labels in shopping for checking proscribed ingredients and other dietary information on food labels (reported by adolescents) *(F)*	Family motivation<br>The importance of parental responsibility in encouraging children to eat healthier foods (by parents) *(F)*	Food preparation and availability<br>Lay explanations of variety and not overindulging guiding their approach to a healthy diet (reported by parents) *(F)*<br><br>Time and cost<br>Using convenience foods such as Halal chicken nuggets and burgers for competing priorities of home and work (reported by parents) *(B)*<br><br>Parenting style<br>Regular family meals with healthy food choices (reported by both) *(F)*<br><br>Fathers treating of either traditional or nontraditional foods, often linked to fast foods or takeaways (reported by both) *(B)*<br><br>Parental practical knowledge and attitudes<br>The concept of a balanced diet or lifestyle being not well conveyed (reported by parents) *(B)*
Rodriguez-Perez et al, 2019, Puerto Rico [73]<br><br>To identify barriers that prevent healthy eating practices in Puerto Rican early adolescents (EAs).	FG<br><br>Underserved community in both urban and rural settings<br><br>Reported by related adolescents and parents/care givers separately<br><br>Content analysis	N = 52 (17)<br><br>Aged 12-14 years<br><br>Female = 67% (76%)<br><br>Purposive sampling of sites by socioeconomic status		Family motivation<br>Unhealthy food intake preferences in the family reducing their will-power to improve healthy food consumption (reported by adolescents) *(B)*	Time and cost<br>Economic cost of eating healthy and the great accessibility of unhealthy food places (reported by both) *(B)*<br><br>Parenting style<br>Providing and rewarding children with candies at home (reported by both) *(B)*<br><br>Parental practical knowledge and attitudes<br>Access to recipes adapted to healthier food choice (reported by both) *(F)*<br><br>Wrongly considering processed rice, flour tortillas, and pastas to be healthy, and not knowing the substantial health benefits of changing processed for whole-grain rice and cereals (reported by both) *(B)*

Roth-Yousey, 2012, US [74]<br><br>To understand parent beverage expectations for early adolescents (EAs) by eating occasion at home and in various settings	FG<br><br>School and community settings in low-income neighborhoods<br><br>Reported by parents/ caregivers<br><br>Not specified	N = (49)<br><br>Aged 10-13 years<br><br>Female = (86%)<br><br>Purposive sampling by BMI		Family motivation<br>Parents' poor role modelling interfering with managing expectations for intake of healthy beverages by their children *(B)*	Food preparation and availability<br>Controlling home availability of unhealthful beverages *(F)*<br><br>Time and cost<br>Increase accessibility to more healthful options, e.g. traditional healthful beverages included water or milk *(F)*<br><br>Improving likeability and accessibility of water by making it cold, flavored, or portable *(F)*
Sedibe et al, 2014, South Africa [42]<br><br>To investigate the narratives pertaining to dietary and physical activity practices by female adolescents in Soweto.	Duo DI<br><br>School setting<br><br>Reported by adolescents<br><br>Thematic analysis	N = 58<br><br>Aged 15.3-21.6 years (mean = 18)<br><br>Female = 100%<br><br>Voluntary response sampling			Food preparation and availability<br>Vegetables not always available in the household *(B)*<br><br>Time and cost<br>Household members often ate kotas as an alternative to home-cooked food as they were "cheaper, convenient, easily accessible and filling" *(B)*
Sharif-Ishak et al, 2020, Malaysia [75]<br><br>To explore the concepts of healthy eating and to identify the barriers	FG<br><br>School setting<br><br>Reported by adolescents	N = 72<br><br>Aged 13-14 years<br><br>Female = 48.6%<br><br>Randomly	Nutrition education<br>Parents as a source of their knowledge, in which parents told them that junk foods contain lots of monosodium glutamate, which is not good for the brain *(F)*	Family motivation<br>Adolescents following their siblings in consuming unhealthy foods *(B)*	Food preparation and availability<br>Availability of food at home as they have to consume what their parents prepared for them regardless if the food was healthy or not so healthy *(F/B)*<br><br>Parenting style<br>Parents' control on adolescents' food choices *(F)*

and facilitating factors for dietary behaviour change in adolescents.	Thematic analysis	selected schools followed by voluntary response sampling			
Silva et al, 2015, Brazil [76]<br><br>To explore how adolescents at a school in the interior of the State of Pernambuco, Brazil, perceive healthy eating	DI<br><br>School setting in low HDI, agricultural region<br><br>Reported by adolescents<br><br>Lexical analysis	N = 40<br><br>Aged 10-14 years<br><br>Female = 62.5%<br><br>Purposive sampling		Family motivation<br>Reception of incentives from family environment through dietary education practices *(F)*<br><br>Lack of time and excessive dedication to work being aspects that interfere in the family's participation in the process of encouraging healthy eating *(B)*	Food preparation and availability<br>Fruit consumption mainly among adolescents who brought the school snack from home or had this meal at home *(F)*<br><br>Time and cost<br>Limited variety of greens and vegetables the adolescents consume during lunch due to their higher cost and their taste *(B)*<br><br>Most subjects' low economic level and the lower cost of nutritionally inappropriate foods rich in sugar, fat and salt favoring the consumption of these foods *(B)*<br><br>Parenting style<br>Mothers spending longer time on family situations, involving aspects of eating, reducing the adolescents' autonomy regarding their dietary choices *(F)*
Siu et al, 2019, Hong Kong [35]<br><br>To investigate the barriers to adopting healthy eating habits among secondary	FG<br><br>School setting in a low-income district<br><br>Reported by adolescents	N = 30<br><br>Secondary 1 and 4<br><br>Female = 50%<br><br>Purposive sampling by gender,			Food preparation and availability<br>Eating at home was associated with a healthy diet under parental influence *(F)*<br><br>Time and cost<br>The financial constraints of the families experienced by the participants normalizing their irregular dining schedules and thus their meal skipping *(B)*

school students from low-income families in Hong Kong	Thematic content analysis	lunchbox practice, and CSSA assistance			Parents being forced to prepare unhealthy food, e.g. fatty meat and preserved vegetables, due to financial strain *(B)*<br><br>Perceiving money as the chief barrier to having healthy restaurant food *(B)*<br><br>Parental practical knowledge and attitudes<br>Parents of some participants incorrectly estimating the nutritional requirements of the family members and unintentionally preparing an imbalanced diet, e.g. inadequate quantities of vegetables *(B)*
Snethen et al, 2007, US [77]<br><br>To understand one Latino community's perspectives about childhood overweight within this high-risk ethnic group	FG<br><br>Community setting<br><br>Reported by unrelated adolescents and parents<br><br>Thematic analysis	N = 12 (24)<br><br>Aged 10-12 years<br><br>Female = 33% (50%)<br><br>Convenience sampling			Food preparation and availability & Time and cost<br>Lack of time for food preparation as a constraint for making healthy children's lunches, after-school snacks, and meals, resulting in eating easy fast food *(B)*<br><br>Parenting style<br>Parents using sweets to reward children and to get them to behave *(B)*
Steeves et al, 2016, US [78]<br><br>To provide in-depth information on the social roles that youths' parents and friends play related to eating and	DI<br><br>Community setting in low-income neighbourhoods<br><br>Reported by related adolescents and parents	N = 38 (10)<br><br>Aged 9 - 15 years<br><br>Female = 42% (80%)<br><br>Purposive sampling by genders, ages and	Nutrition education<br>Intergenerational information exchange from grandparents, e.g. type of foods that were appropriate for youths to consume, creating an expectation for nutrition and health information, passing down cooking skills (reported by both) *(F)*	Family motivation<br>Parents, grandparents and other family members providing support for changing eating behavior (reported by youth) *(F)*<br><br>Parents' unhealthy habits serving as motivation for children to take up healthy habits (by adolescents) *(F)*<br><br>Behavioural modeling among siblings (reported by both) *(F)*	Food preparation and availability<br>Managing the home food environment by modifying their food purchasing behaviours (reported by parents) *(F)*<br><br>Time and cost<br>Cost of grocery items (reported by parents) *(B)*<br><br>Parenting style<br>Creating healthy promoting rules, e.g. having a vegetable with every dinner meal, reducing juice consumption by drinking water between glasses

physical activity behaviours and to explore the impact of other social relationships on youths' eating and physical activity behaviours.	separately<br><br>Direct content analysis	neighbourhood locations			of juice (reported by both) *(F)*<br><br>Parental practical knowledge and attitudes<br>Lack of information regarding the healthfulness of certain items (reported by parents) *(B)*
Tiedje et al, 2014, USA [79]<br><br>To describe the meanings of food, health and wellbeing through the reported dietary preferences, beliefs, and practices of adults and adolescents from four immigrant and refugee communities in the Midwestern United States.	FG<br><br>Immigrant and refugee communities<br><br>Reported by adolescents<br><br>Content analysis and grounded theory	N = 73<br><br>Aged 11-18 years<br><br>Female = 53%<br><br>Purposive sampling by age and gender		Family motivation<br>As a role model to positively influence their parents' eating practices, which they described as unhealthy *(F)*<br><br>Adolescent echoing parents' concerns about staying and eating healthy through balanced meals at home *(F)*<br><br>Cultural belief in the importance of big portion sizes and the image that "a bigger person is healthier" and "a skinny person is a sick person" *(B)*	Food preparation and availability<br>Importance of homemade and fresh food at home, distinguishing it from fast food and junk food in the U.S. *(F)*<br><br>Parents serving them unhealthy ethnic food (i.e. greasy, in large portion size) from their home country because they are familiar *(B)*<br><br>Easy access to junk food at home *(B)*<br><br>Time and cost<br>Parents being "lazy" to prepare food at home and would give them money to eat out *(B)*<br><br>Work schedule impeded dedicating enough time to preparing healthy food *(B)*<br><br>Parents having limited financial ability to provide healthy food for them, e.g. vegetables *(B)*<br><br>Parenting style<br>Intergenerational variation in taste in the same family as a barrier to eating healthy for youth who professed wanting to change to a diet based on fruit and vegetables and low in carbohydrates and fat *(B)*

Verstraeten et al, 2014, Ecuador [80]<br><br>To identify factors influencing eating behaviour of Ecuadorian adolescents - from the perspective of parents, school staff and adolescents - to develop a conceptual framework for adolescents' eating behaviour.	Semi-structured FG<br><br>School setting in low- and middle-income countries<br><br>Reported by unrelated adolescents, parents and school staff<br>Deductive thematic content analysis	N = 80 (32)<br><br>Aged 11-15 years (mean = 41.2 years)<br><br>Female = 52.1% (75%)<br><br>Convenience sampling		Family motivation<br>Negative role modeling on unhealthy eating (reported by parents) *(B)*<br><br>Parents not recognizing their responsibility for their children's dietary behavior, but placed it with school, the environment or their children themselves (reported by parents) *(B)*	Food preparation and availability<br>Availability of healthy food at home by own food production among rural parents and availability in shops among urban parents (reported by both) *(F)*<br><br>Time and cost<br>Adolescents not receiving any/much money and mostly taking food from home to eat at school among low SE groups (reported by parents) *(F)*<br><br>Having less time to prepare (healthy) meals, challenges of organizing their schedules around family meals, and choosing convenient ready-to-eat dishes which are preferred over ''healthy foods'' (by parents) *(B)*<br><br>Cost of healthy food (reported by both from low SE schools) *(B)*<br><br>Parenting style<br>Giving in and adapting meals to children's wishes due to constant struggle to encourage their children to eat healthily by negotiation (by both) *(B)*<br><br>Financial autonomy from pocket money received from parents/grandparents or money earned by them to purchase foods of poor nutritional quality at school (reported by adolescents) *(B)*
Zhang et al, 2018, USA [81]<br><br>To explore Latino fathers' perspectives and parenting experiences regarding	FG<br><br>Community setting<br><br>Reported by fathers	N = (26)<br><br>Aged 10-14 years (33-53 years)<br><br>Female = (0%)	Nutrition education<br>Parental education on healthy eating including talking about the benefits of good nutrition and health properties of foods, teaching cooking skills and letting children watch food related cartoons and	Family motivation<br>Setting expectation for their children to eat fruits, vegetables, appropriate portions of foods, and traditional foods and to finish eating food to avoid waste *(F)*<br><br>Role modeling by setting an example for how they want their children to eat, which included eating vegetables and not	Food preparation and availability<br>Planning, buying, and/or preparing foods for healthy home food environment *(F)*<br><br>Time and cost<br>Encouraging their children to eat fruits and vegetables by strategies such as "cut up" *(F)*<br><br>Resorting to "fast, simple, easy, quick and go"

early adolescents' eating, physical activity, and screen-time behaviours using the focus group method.	Grounded theory	Convenience sampling	documentaries *(F)*<br><br>Child involvement<br>Eating with the children for teaching, monitoring and setting example *(F)*	consuming fast food and sugary drinks *(F)*<br><br>Mutual support and agreement between parents or primary caregivers regarding attitudes, rules, decisions, and communication *(F)*	foods due to busy schedules and getting home from work too tired to prepare more complex meals *(B)*<br><br>Financial constraints preventing their families from being able to afford healthy diets, e.g. organic foods *(B)*<br><br>Parenting style<br>Offering incentives, such as chocolate, pizza, and screen time, to persuade their children to eat certain foods or to play outside *(F/B)*<br><br>Involving their children in food preparation by having them wash, cut, and peel fruits and vegetables (F)<br><br>Observing and controlling portions and frequency of consumption of both healthy and less healthy food, e.g. sugary drinks, sweets, salty snacks, and fast food *(F)*<br><br>Being responsive to a child's preferences, e.g. getting them to eat vegetables based on serving what was liked *(F)*<br><br>Applying pressure regarding expectations "doesn't lead to anything" because they "rebel," "clash," or "get even" at school *(B)*<br><br>Parental practical knowledge and attitudes<br>Encouraging their children to eat fruits and vegetables by strategies such as "vary the vegetables," and "make smoothies" *(F)*

Notes: DI = in-depth interview; FG = focus group; SES = socioeconomic status; B = barrier, F = facilitator
